# A Highly Selective Perylenediimide-Based Chemosensor: “Naked-Eye” Colorimetric and Fluorescent Turn-On Recognition for Al^3+^

**DOI:** 10.3389/fchem.2020.00702

**Published:** 2020-09-11

**Authors:** Yan Liu, Shuang Gao, Liu Yang, Yu-Long Liu, Xiao-Min Liang, Fei Ye, Ying Fu

**Affiliations:** Department of Applied Chemistry, College of Arts and Sciences, Northeast Agricultural University, Harbin, China

**Keywords:** fluorescence, perylened derivatives, Al^3+^, turn on, hydrolysis

## Abstract

A novel “turn-on” fluorescent probe (**PCN**) was designed, synthesized, and characterized with perylene tetracarboxylic disimide as the fluorophore and Schiff base subunit as the metal ion receptor. The probe demonstrated a considerable fluorescence enhancement in the presence of Al^3+^ in DMF with high selectivity and sensitivity. Furthermore, the considerably “off–on” fluorescence response simultaneously led to the apparent color change from colorless to brilliant yellow, which could also be identified by naked eye easily. The sensing capability of **PCN** to Al^3+^ was evaluated by the changes in ultraviolet–visible, fluorescence, Fourier transform–infrared, proton nuclear magnetic resonance, and high-resolution mass spectrometry spectroscopies. The linear concentration range for Al^3+^ was 0–63 μM with a detection limit of 0.16 μM, which allowed for the quantitative determination of Al^3+^.

## Introduction

Aluminum is the third most prevalent metal in the lithosphere, and it is toxic to living organisms due to its potential neurotoxicity in excessive amounts (Frantzios et al., 2000; Abeywickrama et al., 2019). Excess Al^3+^ will induce a wide range of diseases such as microcytic hypochromic anemia, osteoporosis, muscular atrophy, and Alzheimer's disease (Cronan et al., 1986; Fasman, 1996; Nayak, 2002; Walton, 2007; Zhang et al., 2020). According to the recommendation of the World Health Organization, the tolerable value of average human intake of Al^3+^ ions is around 3.0–10.0 mg/day (Valeur and Leray, 2000; Qin et al., 2014). Moreover, the environment may be polluted due to high level of Al^3+^ in the ecosystem (Godbold et al., 1988; Sade et al., 2016; Ye et al., 2019a). Fluorescence techniques for detecting various ions have become an optimal choice due to their high sensitivity and selectivity (Suresh et al., 2018; Ye et al., 2019b; Bai et al., 2020; Wu et al., 2020; Zhao et al., 2020). In the past few years, various fluorescent chemosensors including coumarin, naphthalimide, pyrene, BODIPY, anthracene, and rhodamine were exploited for detection of Al^3+^ (Samanta et al., 2014; Hossain et al., 2017; Kozlov et al., 2019; Prabhu et al., 2019; Roy et al., 2019; Li et al., 2020).

Perylene tetracarboxylic disimide derivatives (PDIs), as a representative of strong fluorescence and superior functional organic dyes, display exceptional optical, redox, and electrochemical properties and high thermal stability (Ahrens et al., 2003; Wurthner, 2004; Wasielewski, 2006; Xu et al., 2008; Luo and Chen, 2013; Gao et al., 2020). In addition to their industrial application as pigment, many PDIs also exhibit unique spectroscopical, near-unity fluorescence quantum yields, and strong electron-deficient nature. Owing to the delocalization effect and rigid plane, PDIs are endowed with high fluorescence quantum yields and have been widely utilized as a chromophore (Miyake et al., 2006; Soh et al., 2006; He et al., 2007; Kirschning et al., 2007; Yan et al., 2009; Ruan et al., 2010; Nanashima et al., 2012; Zhang et al., 2013). Fluorescent chemosensors based on modified perylene dye have been used to monitor various ions. PDI-based 2-thiophenamine derivative was reported for the selective determination of Hg^2+^ with a detection limit of 2.2 μM in DMF–H_2_O (1:1, *v*/*v*; Malkondu and Erdemir, 2015). A new water-soluble fluorescent probe was given by the condensation polymerization between 2,2′:6′,2″-terpyridine-containing dibromide and perylene diimide–diamines, which was used to determine Fe^3+^ and monitors the Fe^3+^/Fe^2+^ transition after the addition of a reducing agent such as ascorbic acid (Vc; Jin et al., 2016).

**Scheme 1:**
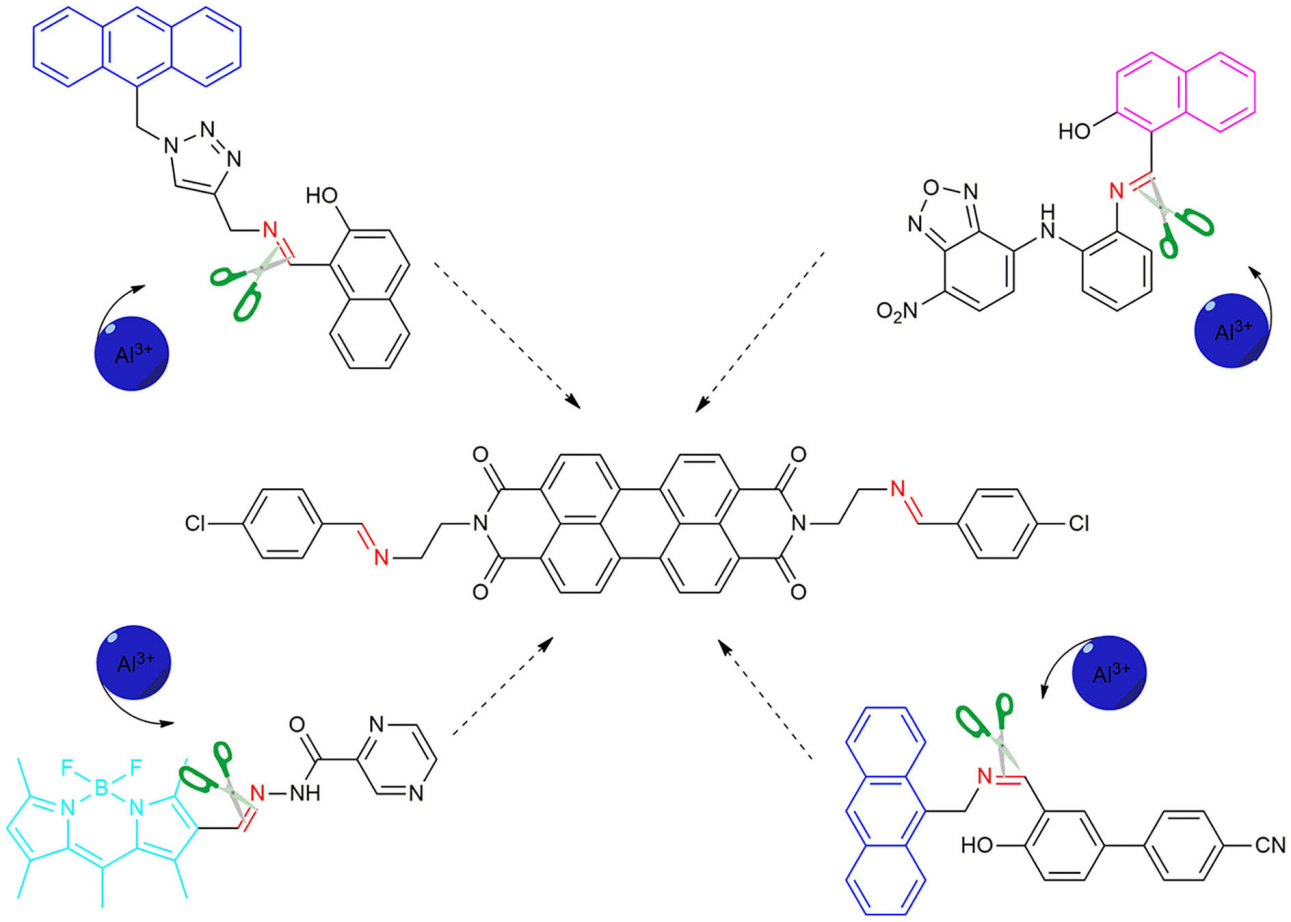
The design of the fluorescent probe **PCN** for Al^3+^.

**Scheme 2:**
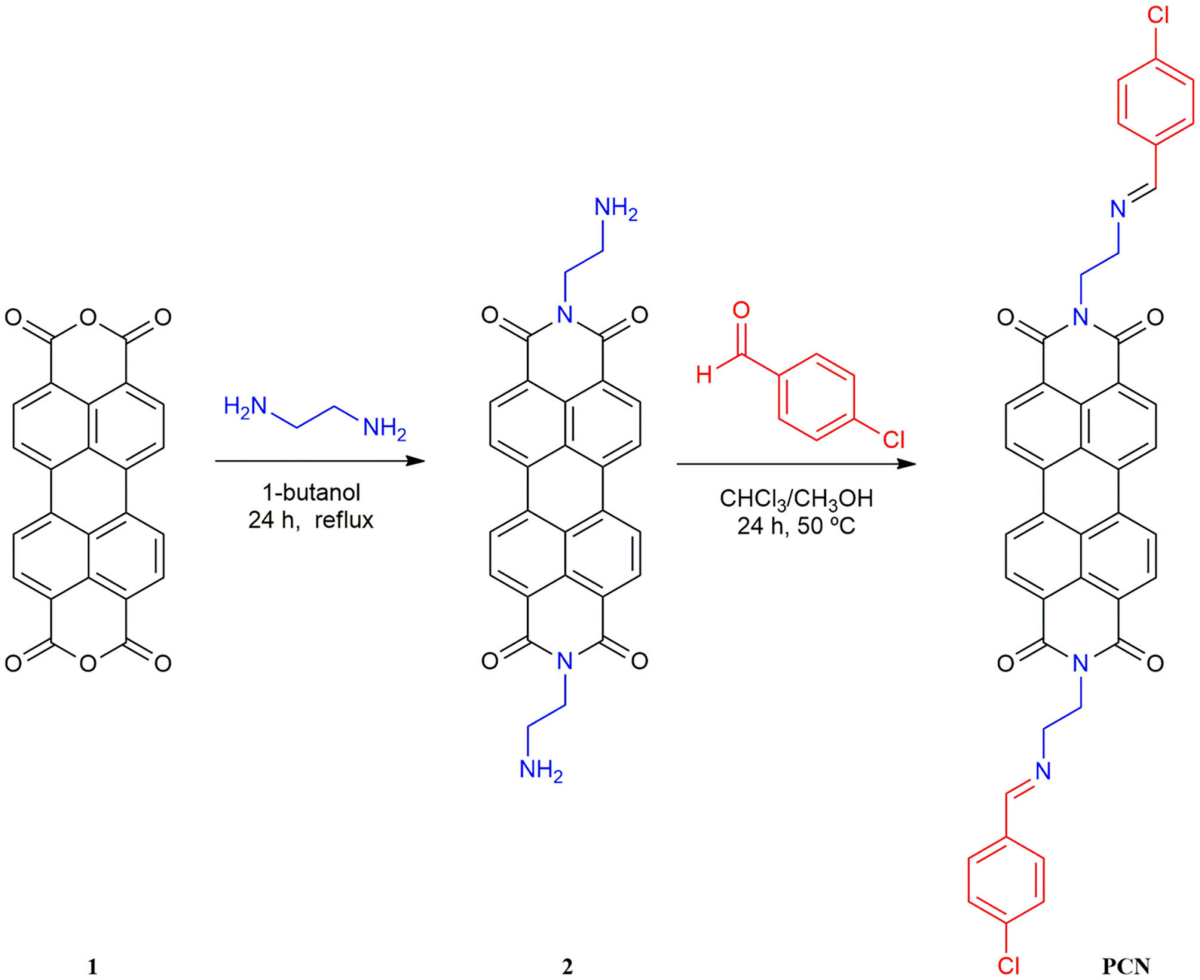
Synthesis of perylene-3,4,9,10-tetracarboxylic diimide probe **PCN**.

Herein, a novel perylenebisimide-based fluorescent sensor for monitoring Al^3+^ had been designed (Scheme 1; Erdemir and Kocyigit, 2018; Kashyap et al., 2019; Kumar et al., 2019; Erdemir et al., 2020). *N,N*′-bis[(2-*p*-chlorobenzaldehyde)-ethyl] perylene-3,4,9,10-tetracarboxylic diimide (**PCN**) was obtained via the polycondensation reaction of amino functionalized perylene-3,4,9,10-tetracarboxylic diimide and *p*-chlorobenzaldehyde (Scheme 2) and characterized by Fourier transform–infrared (FT-IR), proton nuclear magnetic resonance (^1^H NMR), carbon-13 nuclear magnetic resonance (^13^C NMR), and high-resolution mass spectrometry (HRMS) spectroscopies. It showed drastic fluorescence enhancement and obvious color change toward its binding of Al^3+^ in DMF solution with excellent selectivity and sensitivity.

## Experimental

### Materials and Instruments

All chemicals and solvents were purchased from commercial providers and used without purifying. FT-IR spectra were measured using a Bruker ALPHA-T spectrometer (KBr, Bruker, Germany). The ^1^H NMR and ^13^C NMR spectra were recorded on a Bruker AVANVE 400 MHz system (Bruker, Germany) using CF_3_COOD as the solvent. The HRMS was carried out on an FTMS Ultral Apex MS spectrometer (Bruker Daltonics Inc., USA). The ultraviolet–visible (UV-vis) spectra were gained on a UV-2550 ultraviolet spectrophotometer (Shimadzu, Japan). The fluorescence spectra were obtained through the PerkinElmer LS55 fluorescence spectrometer (PerkinElmer, UK). All pH values were made with PHS-3C pH meter (Inesa, China).

### Synthesis of *N,N′*-bis(ethylenediamine)Perylene-3,4,9,10-Tetracarboxylic Bisimide (2)

The *N,N*′-bis(ethylenediamine)perylene-3,4,9,10-tetracarboxylic bisimide (**2**) was synthesized according to the reported literatures (Cheng et al., 2009; Chang et al., 2012). A solution of ethylene diamine (3.0 g, 10 mmol) and perylene-3,4,9,10-tetracarboxylic acid dianhydride (**1**; 1.0 g, 0.5 mmol) in 1-Butanol (100 mL) was refluxed with continuous stirring at 90°C. After refluxing for 24 h, the resulting mixture was treated with 0.2 M sodium acetate–acetic acid buffer solution (pH = 5.3, 50 mL). Then, 1 M NaOH was added to the filtrate until pH = 13. The residue was filtered and washed with water and methanol, and dried to give compound **2**. Yield: 46 %. m.p. > 300°C. All spectra of structural characterization of compounds are in the Supporting Information. FT-IR data in KBr (cm^−1^): 3,360, 3,296 (*v* N-H), 2,933, 2,894, 2,841 (*v* C-H), and 1,674(*v* C=O). ^1^H NMR [δ_H_ in parts per million (ppm) in CF_3_COOD (TMS), 400 MHz]: 8.94–9.01 (m, 8H, Ar-H), 4.96 (br, 4H, -CH_2_-), and 3.98 (br, 4H, -CH_2_-). ^13^C NMR [δ_C_ in CF_3_COOD (TMS), 100 MHz]: 166.38, 136.31, 133.09, 129.33, 126.33, 124.34, 121.56, 116.19, 115.15, 113.38, 112.34, 40.57, and 38.40.

### Synthesis of *N,N′*-bis[(2-p-Chlorobenzaldehyde)-ethyl]Perylene-3,4,9,10-Tetracarboxylic Diimide

The final product **PCN** was synthesized on the basis of the previous literatures (Malkondu and Erdemir, 2015; Fu et al., 2019a). Compound **2** (0.46 g, 1 mmol) was suspended first in MeOH/CHCl_3_ (1:1, 160 mL) followed by adding 0.7 g, 5 mmol *p*-chlorobenzaldehyde, and then the mixture was stirred at 50°C for 48 h. After cooling to room temperature, the precipitated solid was obtained and washed with methanol. The residue was recrystallized from MeOH/CHCl_3_ (*v*/*v*, 4:1) to obtain the dark-red solid. Yield: 84 %. m.p. > 300°C; FT-IR data in KBr (cm^−1^): 2,932, 2,822 (v C-H), 1,682, and 1,646 (v C=O); ^1^H NMR [δ_H_ in ppm in CF_3_COOD (TMS), 400 MHz]:8.99 (s, 2H, CH=N), 8.78 (s, 8H, Ar-H), 7.98–7.96 (d, 4H, Ar-H), 7.69–7.67 (d, 4H, Ar-H), 4.92 (br, 4H, -CH_2_-), and 4.57 (br, 4H, -CH_2_-). ^13^C NMR [δ_C_ in CF_3_COOD (TMS), 100 MHz]: 172.16, 165.69, 147.91, 136.05, 132.93, 132.79, 130.81, 129.10, 126.08, 124.25, 123.74, 121.38, 51.63, and 39.09. HRMS (ESI): calcd. for C_42_H_26_N_4_O_4_Cl_2_ ([M+H]^+^) 721.5862 found 721.1407.

### Spectrophotometric Studies

The stock solution of **PCN** (10^−3^ M) was dissolved in DMF and then diluted to 10^−5^ M for the spectroscopic measurements of Al^3+^. The stock solutions of 10^−2^ mol L^−1^ concentration of metal ions were provided from AlCl_3_, FeCl_3_, CrCl_3_, MgCl_2_, PbCl_2_, ZnCl_2_, Hg(OAc)_2_, CaCl_2_, CuCl_2_, CoCl_2_, MnCl_2_, SnCl_2_, NiCl_2_, BaCl_2_, NaCl, KCl, and AgNO_3_ using ultrapure water. In the selectivity measurement, 10 mL of the **PCN** solution (1 × 10^−5^ M) and 50 μL of each metal ion stock solution (10^−2^ M) were added to volumetric flasks. The probe **PCN** stock solution (10 mL) was mixed with gradual incremental Al^3+^ solution separately for the titration experiments. These resulting solutions were well mixed, and then the spectral properties were recorded after 6 h. The excitation was set at 500 nm for the measurement of fluorescence, and slit width of the excitation was 10 nm. In spectral experiment, the concentration titration was measured at least twice to ensure consistent results. All the measurements were obtained at 25°C.

## Results and Discussion

### UV-Vis and Fluorescence Spectral Characteristics Studies

**Figure 1:**
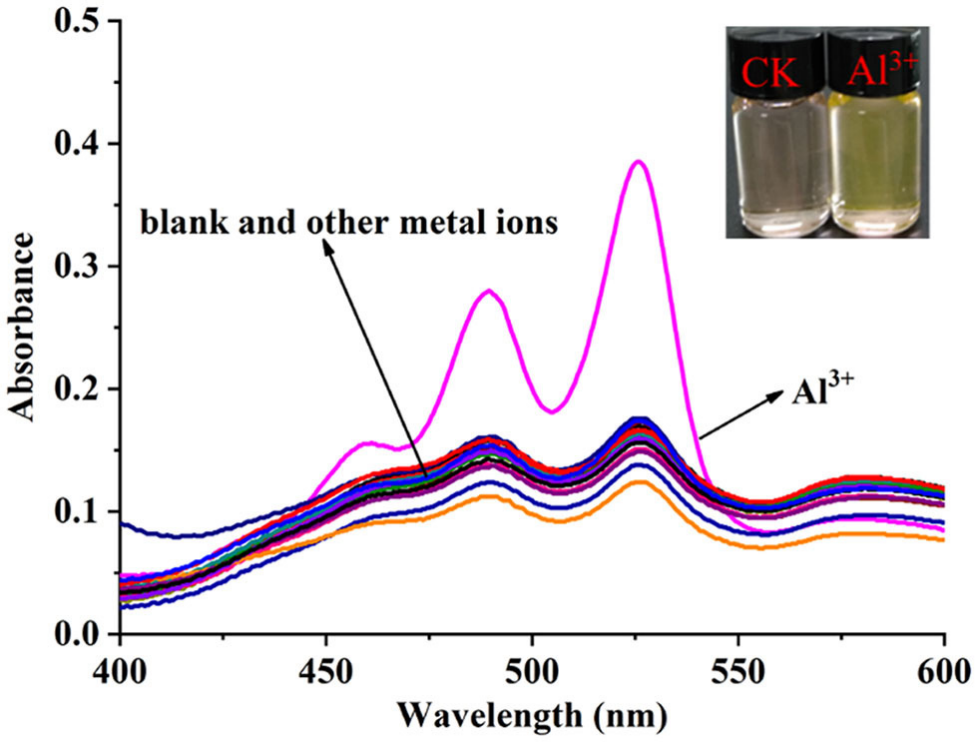
UV-vis spectra changes of the **PCN** solution (10 μM) after different metal ions (50 μM) were added in DMF. Inset: color changes of **PCN** solution before and after adding Al^3+^ (50 μM) under natural light.

**Figure 2:**
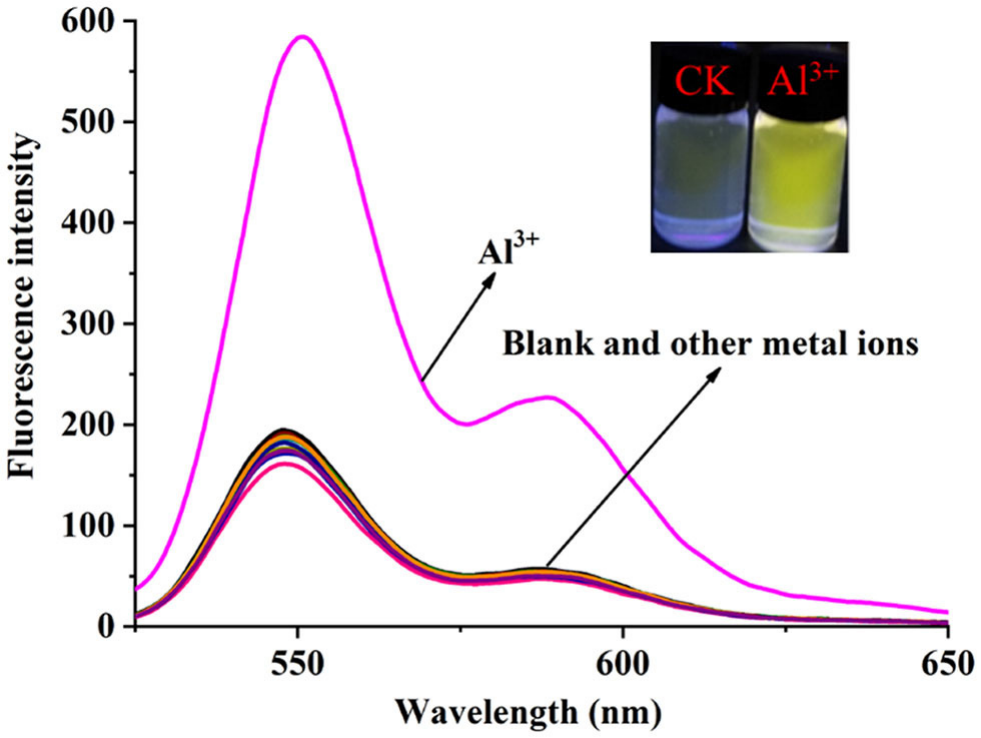
Fluorescence spectra changes of the **PCN** solution (10 μM) after different metal ions (50 μM) were added in DMF (λ_ex_ = 500 nm). Inset: color changes of **PCN** solution before and after adding Al^3+^ (50 μM) under UV light of 365 nm.

The solvent effect of **PCN** has been studied through fluorescence measurement in different solvents (Supplementary Figure 8). The probe exhibits weak fluorescence properties in almost all the investigated solvents except EtOH. The compound exhibits weak yellow fluorescence emission with peaks from 541 nm (MeCN) to 558 nm (DMSO) in most solvents, but much no fluorescence in EtOH was observed. Based on the response mode of the fluorescent molecular probe's “turn on” and “turn off,” the solvent was selected for further study. The probe had failed to show the good selectivity toward various ions in DMSO and other solvents. The photophysical property of the fabricated fluorescent chemosensor in DMF was investigated. Free probe (**PCN**) demonstrated weak fluorescence at about 550 and 590 nm. To estimate the selectivity and sensitivity of **PCN** (10 μM), the UV-vis and fluorescence spectra of **PCN** toward different metal cations (such as Al^3+^, Fe^3+^, Cr^3+^, Mg^2+^, Pb^2+^, Zn^2+^, Hg^2+^, Ca^2+^, Cu^2+^, Co^2+^, Mn^2+^, Sn^2+^, Ni^2+^, Ba^2+^, Na^+^, K^+^, and Ag^+^) have been investigated. UV-vis spectra of **PCN** were obtained in the existence of 5 equiv. of the tested cations. The absorption of **PCN** increased significantly in the presence of Al^3+^ at 490 and 525 nm, and the colorless solution of **PCN** changed to yellow under natural light, indicating that the “naked eye” is visible (Figure 1). The fluorescence responses of **PCN** were measured in the presence of fivefold excess of various metal ions (Figure 2). Upon the addition of different cations (5 equiv.), only when added the Al^3+^ into the solution can it induce a significant fluorescence enhancement at 550 and 590 nm. Moreover, a yellow-colored visual fluorescence change was observed after adding Al^3+^ ion to the **PCN** solution. In contrast, most of the other metal ions, including some mono-, di-, and trivalent metal ions (Ag^+^, Na^+^, K^+^, Mg^2+^, Pb^2+^, Zn^2+^, Hg^2+^, Ca^2+^, Cu^2+^, Co^2+^, Mn^2+^, Sn^2+^, Ni^2+^, Ba^2+^, Cr^3+^, and Fe^3+^), were unresponsive to this system. The imine bond in **PCN** was hydrolyzed by the addition of Al^3+^ due to the Lewis acid character of Al^3+^, and compound **2** with strong fluorescent was released. It resulted to a prominent “light-on” yellow solution and fluorescence emission of **PCN**, which allowed for naked-eye detection of Al^3+^ under natural light and UV light of 365 nm. This mechanism was proven by FT-IR, ^1^H NMR, and HRMS experiments. Therefore, **PCN** showed “off–on” response to Al^3+^ ions in the DMF solution. All these showed the good selectivity of **PCN** toward Al^3+^ over other cations.

### UV-Vis and Fluorescence Titration Experiments

**Figure 3:**
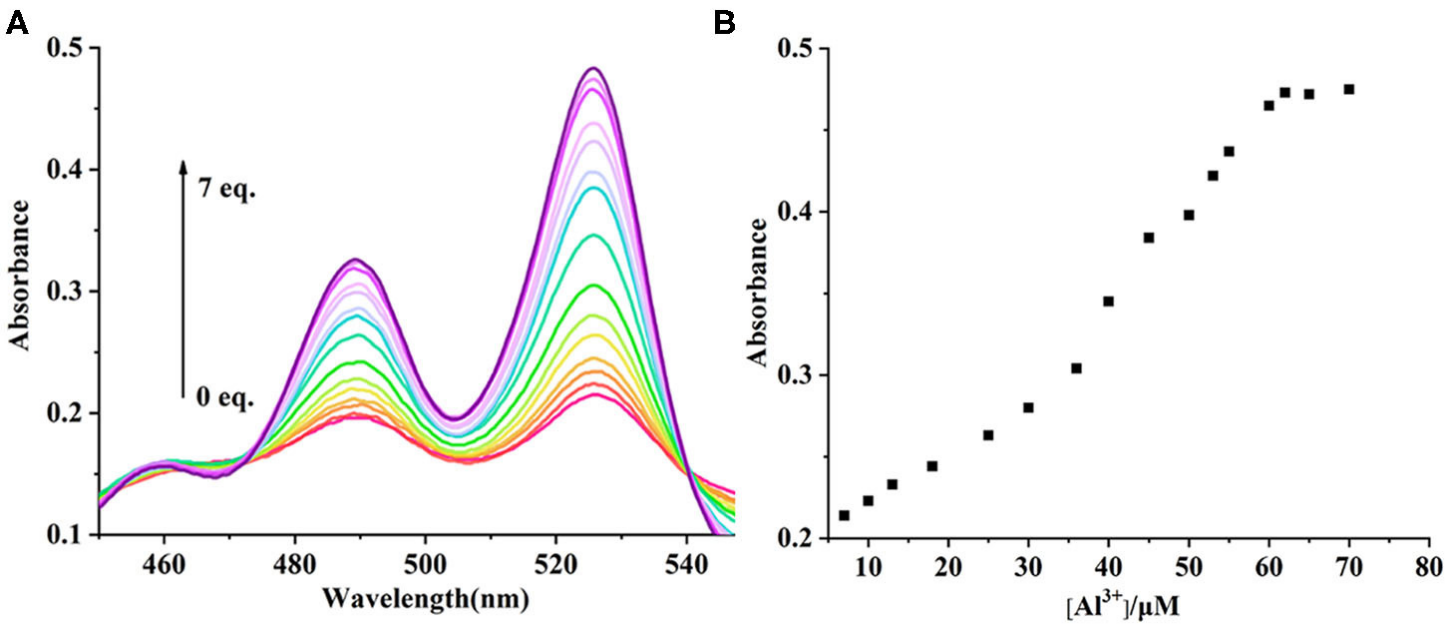
**(A)** UV-vis spectra recorded for probe **PCN** (10 μM) in DMF upon spectrometric titration with Al^3+^ (0–70 μM). **(B)** The relationship between the absorbance (525 nm) and the concentration of Al^3+^.

**Figure 4:**
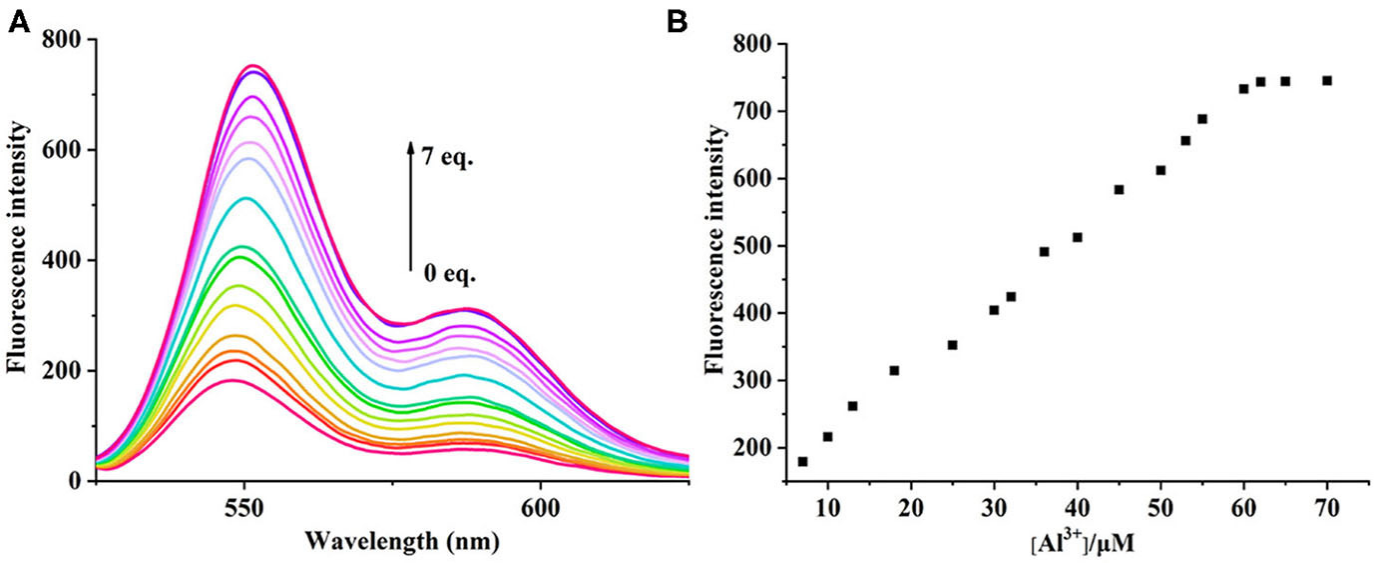
**(A)** Fluorescence spectra recorded for probe **PCN** (10 μM) in DMF upon spectrometric titration with Al^3+^ (0–70 μM). **(B)** The relationship between the fluorescence intensity (550 nm) and the concentration of Al^3+^.

**Figure 5:**
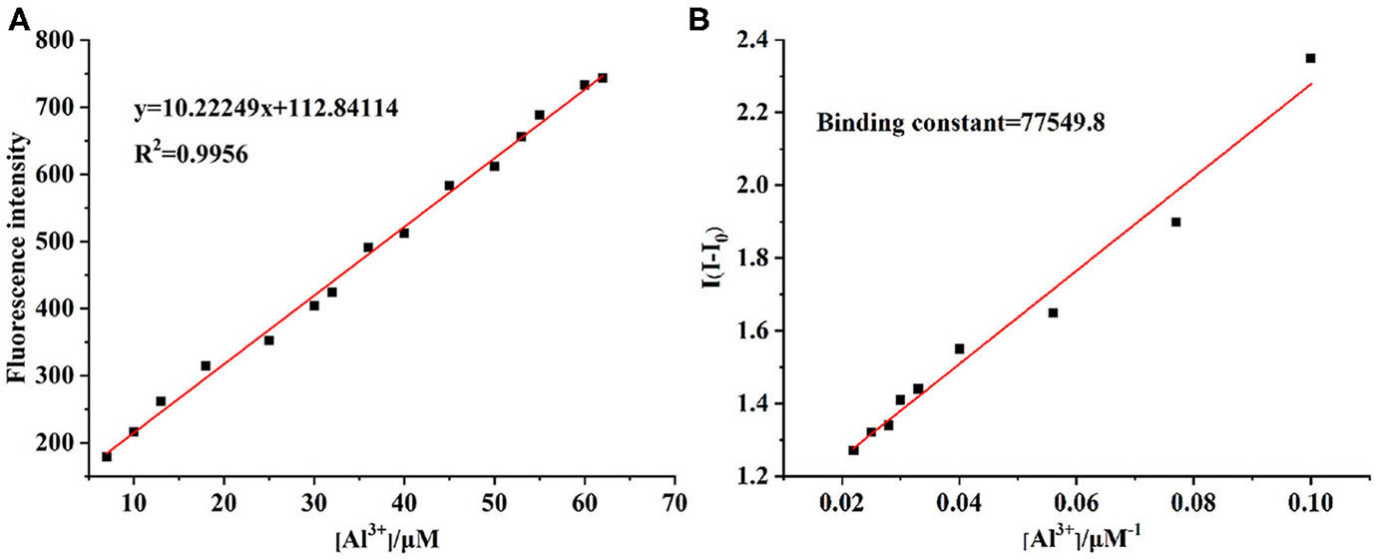
**(A)** The linear responses of fluorescence intensity (550 nm) with Al^3+^ concentrations. **(B)** Benesie–Hildebrand plot of **PCN** between **PCN** and Al^3+^ in DMF.

The UV-vis and fluorescence titrations were measured by increasing the amount of Al^3+^ (0–70 μM) to **PCN** in DMF. The intensity of absorbance at 490 and 525 nm increased gradually after the addition of an increasing amount of Al^3+^ (Figure 3). The absorbance of **PCN** became steady at 525 nm with the Al^3+^ concentration being 63 μM. The change of UV-vis spectral could attribute to the binding affinity of **PCN**. As depicted in Figure 4, the independent **PCN** exhibited a weak emission in DMF. However, a distinct increase in fluorescence emission was observed after Al^3+^ was added and a plateau with the addition of 63 μM of Al^3+^ was achieved at 550 nm. The weak fluorescence may correspond to the photoinduced electron transfer (PET) process resulted from the N atom of imine (C=N) to the luminescent perylene unit (Upadhyay et al., 2018; Fu et al., 2019b). With the increase of Al^3+^, the PET effect of the sensor **PCN** is inhibited, and thereby the intense fluorescence of PDI units is restored. Also, it clearly indicates the structural change of **PCN** by the interaction of Al^3+^ with **PCN**. As shown in Figure 5A, the fluorescence intensity showed a linear relationship (*R*^2^ = 0.9956) with concentration of Al^3+^ in the range of 0–63 μM, indicating that **PCN** could be used to determine the Al^3+^ quantitatively. The detection limit of **PCN** for sensing Al^3+^was 0.16 μM based on applying equation *LOD* = 3σ/*k*, which was calculated from the linear regression curve of the fluorescence intensity and the Al^3+^ concentration. This indicated that **PCN** had high fluorescence selectivity to Al^3+^. In addition, the binding constant, K, could also be obtained from the Stern–Volmer equation *I*_0_/(*I*_0_ − *I*) = 1/*A* + 1/*KA* · 1/[*Q*]. The *K* for Al^3+^ was calculated to be 7.75 × 10^4^ M^−1^ in the DMF solution, as increased from the fluorescence titration curves of probe **PCN** with Al^3+^ (Figure 5B). The comparison of probe **PCN** with other Al^3+^ chemical sensors based on Schiff's base was summarized in Table 1 with different sensing mechanisms (Gan et al., 2017; Roy et al., 2017; Tajbakhsh et al., 2017; Shen et al., 2018; Wang et al., 2018; Zhang et al., 2018; Fu et al., 2019). Compared with other sensors, the advantage of probe **PCN** was its lower detection limit, but its insolubility in water was its shortcoming, which might limit its application in biological and environmental chemistry to some extent.

**Table 1:** Comparison of **PCN** with previously published probes for Al^3+^ ions.

**Probes**	**Working media**	**LOD (μM)**	**Sensing mechanism**	**References**
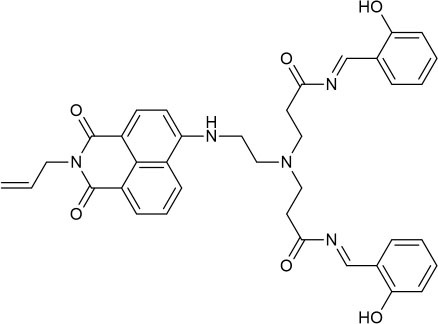	EtOH:Tris (1:1 *v*/*v*)	0.34	PET	Shen et al., 2018
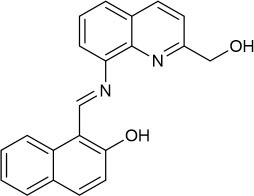	MeOH:H_2_O (8:1 *v*/*v*)	0.1	ICT and CHEF	Fu et al., 2019
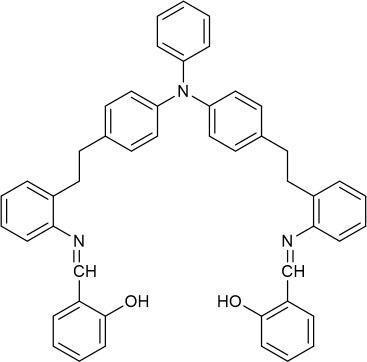	EtOH:H_2_O (4:1 *v*/*v*)	0.299	CHEF	Gan et al., 2017
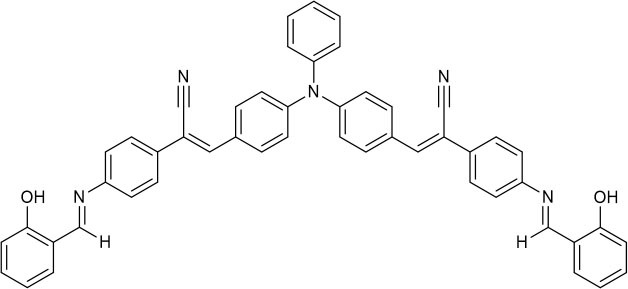	EtOH:H_2_O (6:4 *v*/*v*)	4.369	hydrolysis	Zhang et al., 2018
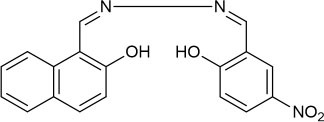	MeOH:H_2_O (9:1 *v*/*v*)	4.39	PET and CHEF	Roy et al., 2017
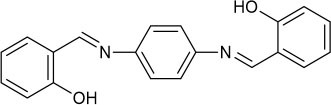	DMF:H_2_O (4:1 *v*/*v*)	0.39	AIE	Wang et al., 2018
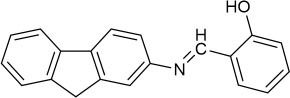	CH_3_CN	0.31	ESIPT and C=N isomerization	Tajbakhsh et al., 2017
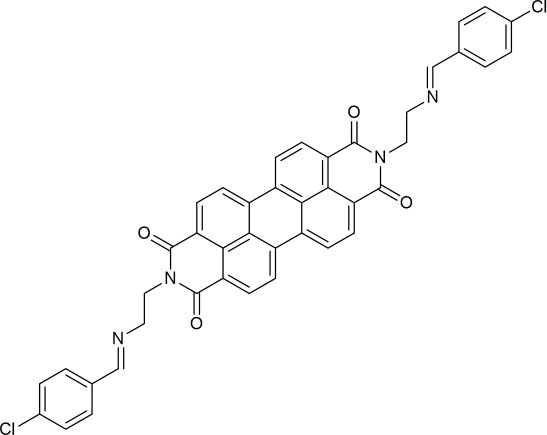	DMF	0.16	hydrolysis	This work

*LOD, limit of detection; ICT, internal charge transfer; PET, photoinduced electron transfer; CHEF, chelation-enhanced fluorescence; AIE, aggregation-induced emission; ESIPT, excited-state intramolecular proton transfer*.

### The Competition Experiments Studies

**Figure 6:**
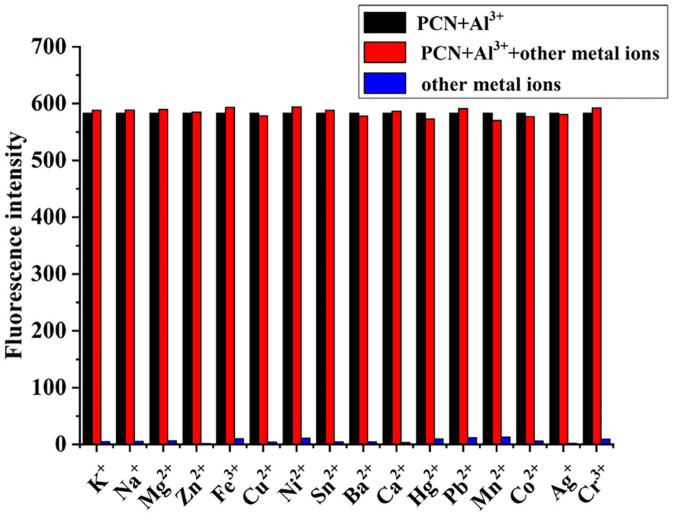
Competition selectivity of **PCN** (10 μM) toward Al^3+^ (50 μM) in the presence of other competition metal ions (100 μM) with emission at 550 nm.

The fluorescence spectra of **PCN** were investigated to examine its selectivity in the existence of other metal ions. First, 10 equiv. of background metal ions (100 μM) was added to the solution of **PCN** (10 μM) to form a **PCN**/M^n+^ system, and then, 5 equiv. of Al^3+^ (50 μM) was added into the solution. As shown in Figure 6, Al^3+^ detection by compound **PCN** was not influenced by the selected background metal cations. Therefore, the combined results clearly indicated that **PCN** exhibited remarkable Al^3+^ signaling behavior and can function as a high selectivity and disturbance-free Al^3+^ fluorescent probe even in the existence of most competing metal cations.

### Sensing Mechanism of PCN for Al^3+^

**Figure 7:**
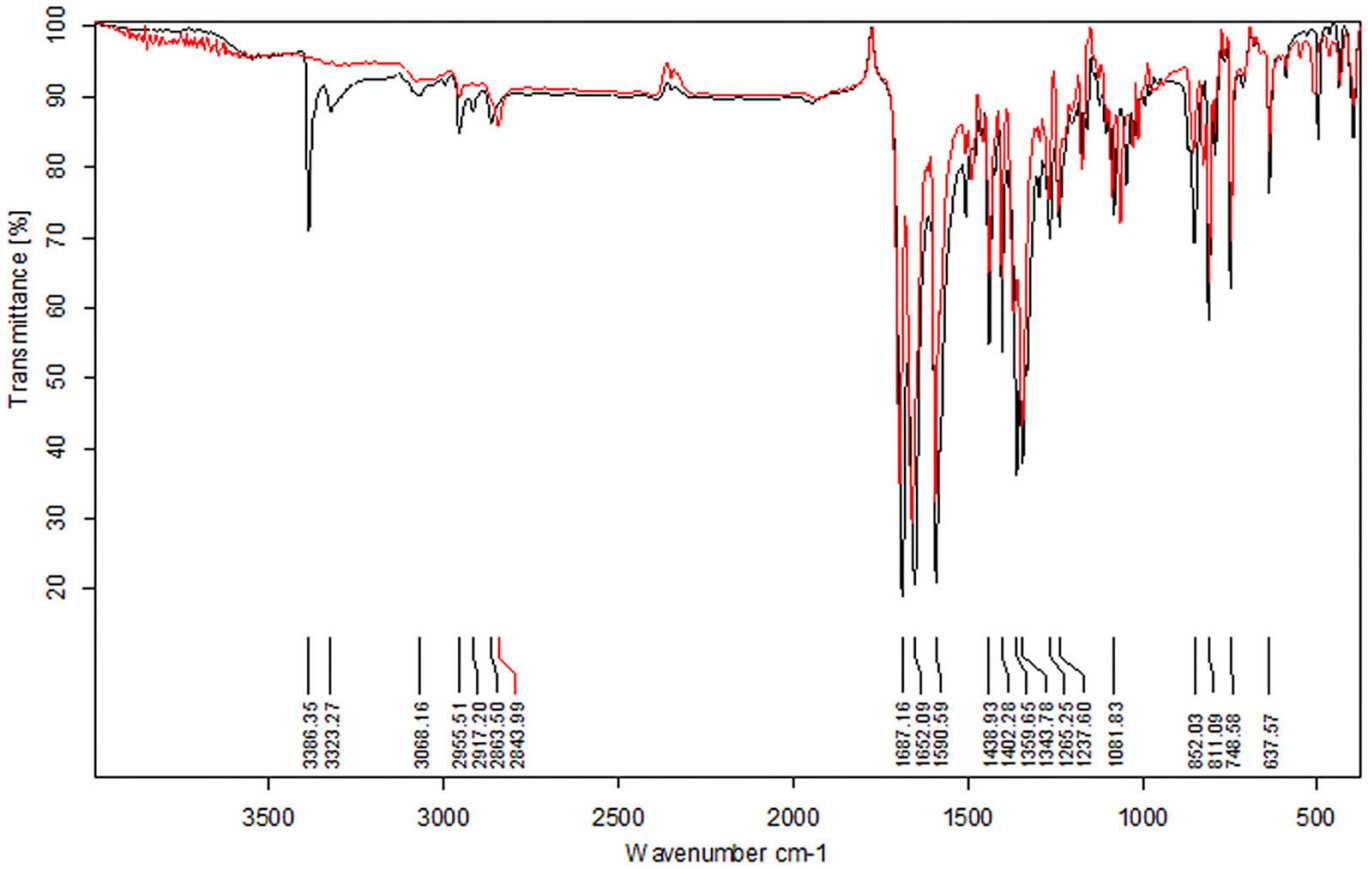
The FT-IR spectra of compound **PCN** before (red) and after (black) the addition of Al^3+^.

**Figure 8:**
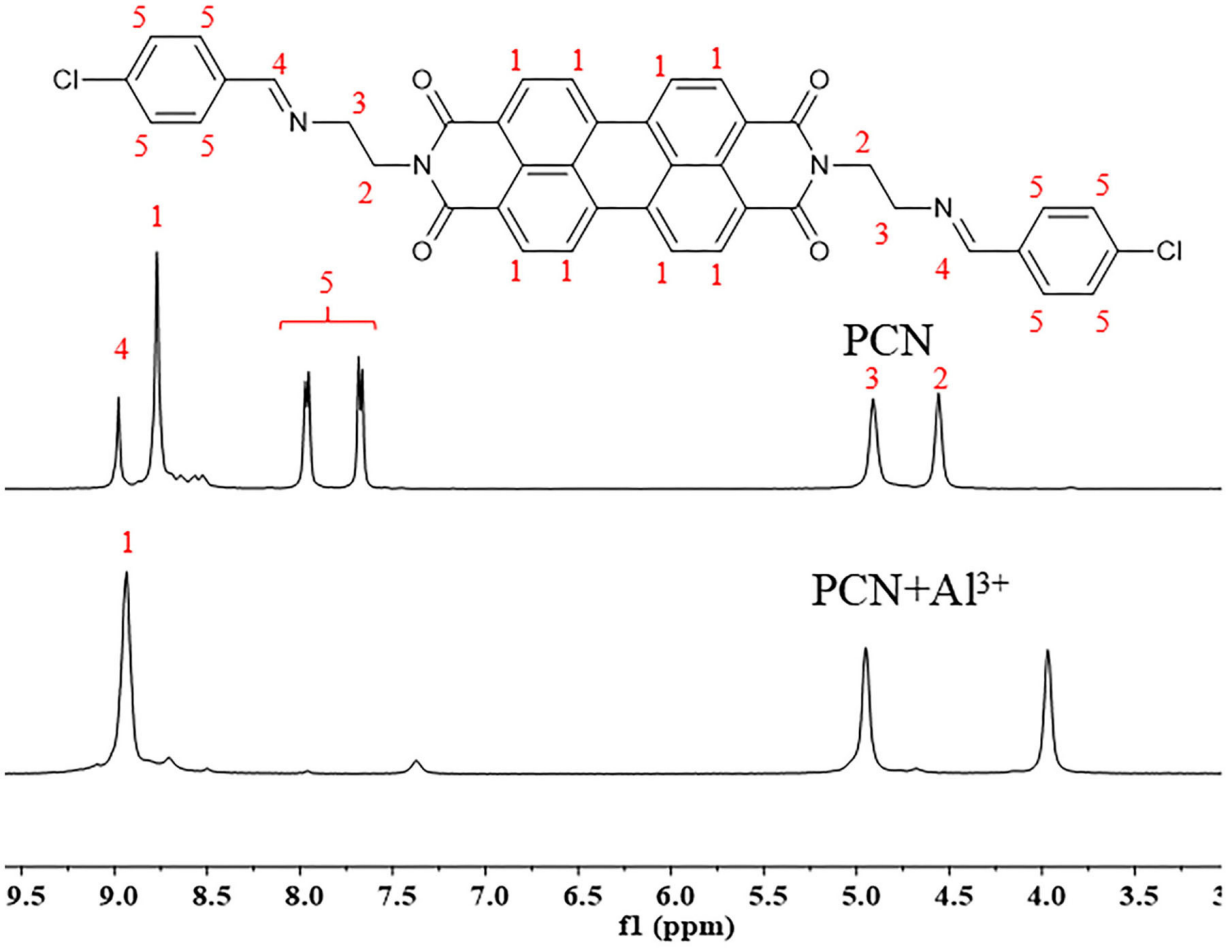
The ^1^H NMR spectra of **PCN** and the isolated product of **PCN** with Al^3+^.

**Figure 9:**
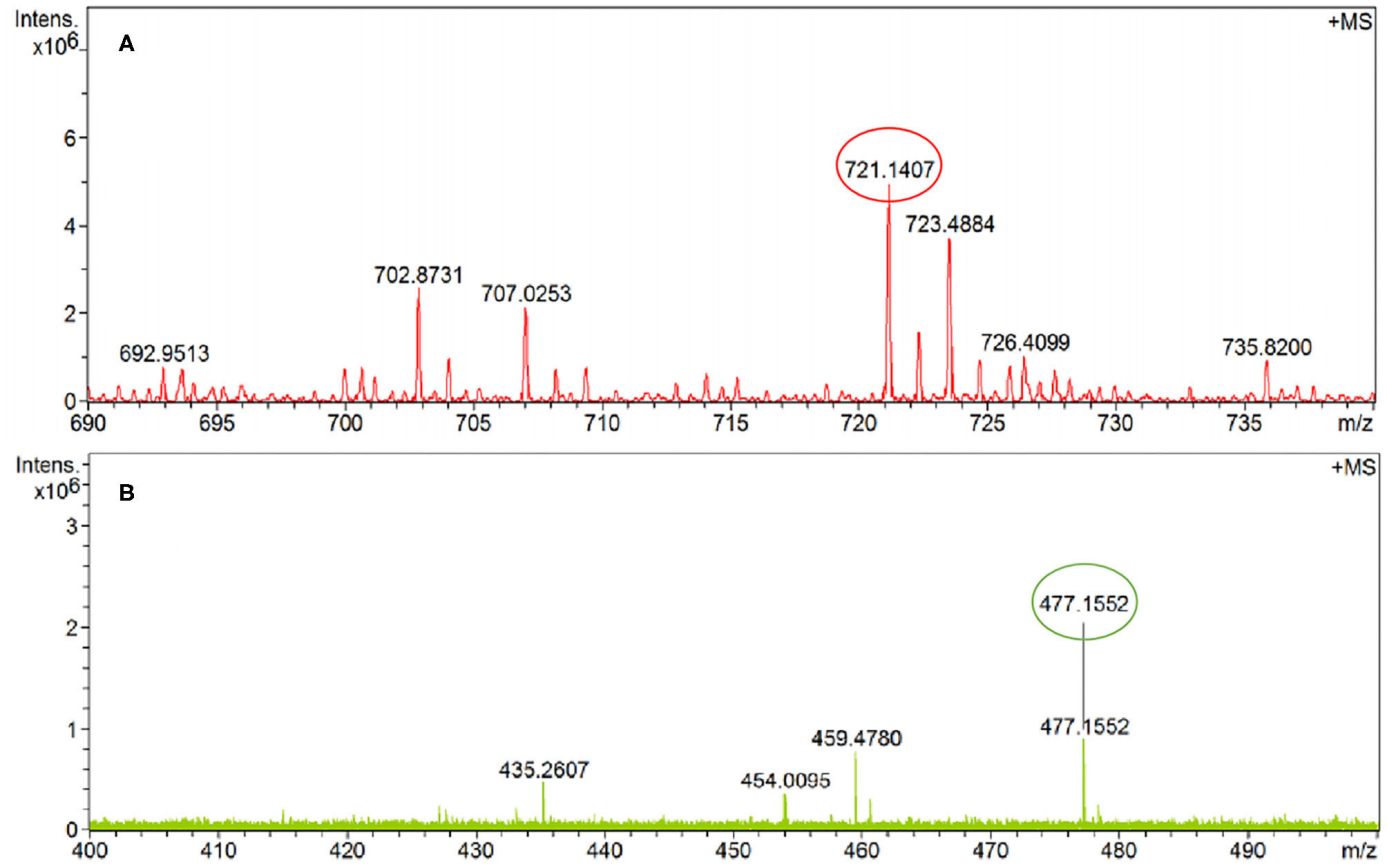
The HRMS spectra of **PCN (A)** and **PCN**–Al^3+^ **(B)**.

**Figure 10:**
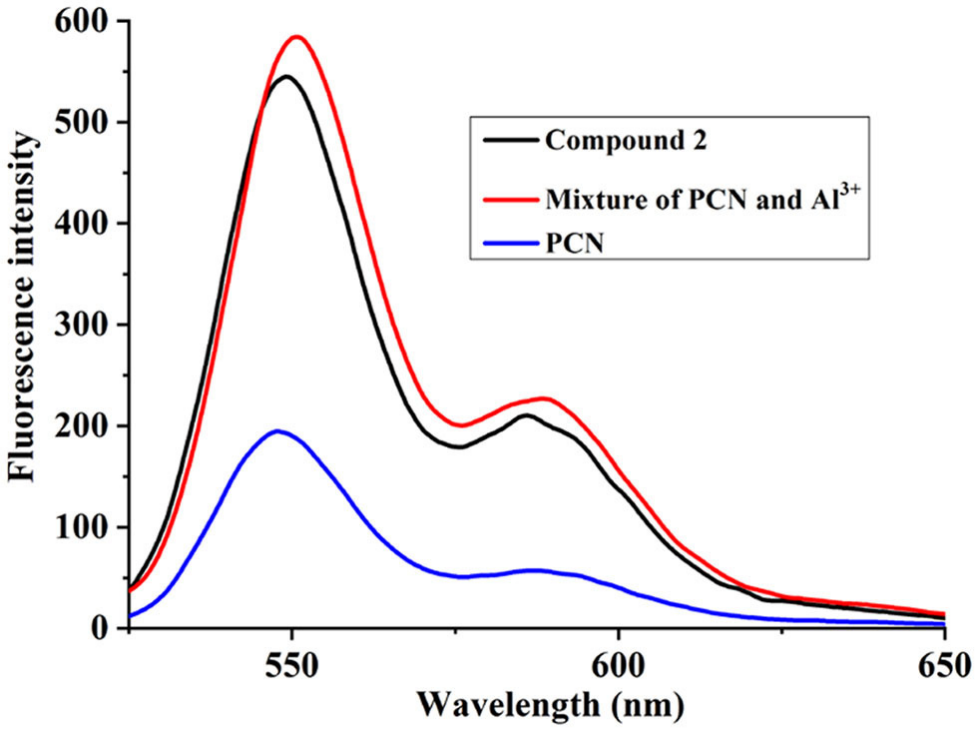
Fluorescence spectra responses of **PCN**, compound **2**, and mixture of **PCN** and Al^3+^in DMF.

**Scheme 3:**
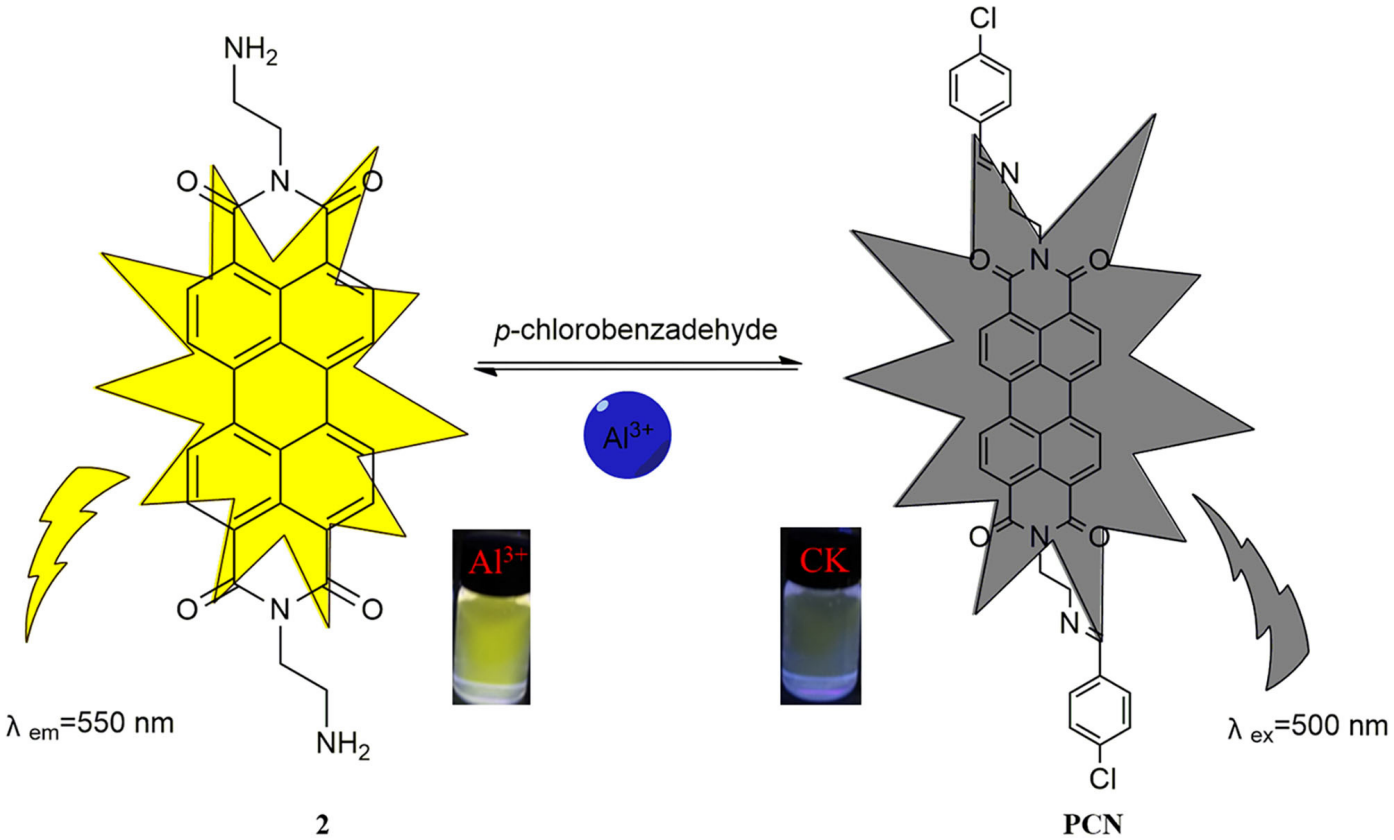
Proposed sensing mechanism toward Al^3+^.

To elucidate the sensing mechanism, the IR, NMR, and HRMS spectra of **PCN**-Al^3+^ were performed. The FT-IR spectroscopic analysis of **PCN** and **PCN**–Al^3+^ complexes is shown in Figure 7. Compared with the FT-IR spectra of **PCN**, the peaks appeared at 3,386 and 3,323 cm^−1^ in the FT-IR spectra after the addition of 5 equiv. of Al^3+^, which is consistent with the amino peak of intermediate **2**. The result suggested that intermediate **2** might be regenerated, which is consistent with the previous spectral analysis. The ^1^H NMR spectroscopic analysis of **PCN** and the reaction product of **PCN** with Al^3+^ are shown in Figure 8. The addition of Al^3+^ resulted in different peak profiles, the signal of aldimine protons (H_4_) at 8.99 ppm completely disappeared, and these peaks at 7.67–7.98 ppm corresponding to aromatic protons (H_5_) also disappeared comparing with ^1^H NMR spectra, which also confirmed that probe **PCN** was hydrolyzed. Moreover, by comparing the HRMS spectra in Figure 9, it was found that the original peak at [M+H]^+^ 721.1407 for free **PCN** disappeared, and a new peak at [M+H]^+^ 477.1552 emerged after the addition of Al^3+^. All results clearly delineated that Al^3+^ induced the cleavage of imine. In order to get full insight into the mechanism, the fluorescence spectra of compound **2** and **PCN** in the absence and presence of Al^3+^ were recorded separately in Figure 10. Upon the spectral changes of **PCN** induced by Al^3+^, it was found that the spectral data were nearly identical with those of compound **2**, which clearly confirmed the cleavage of the C=N of **PCN** in the presence of Al^3+^(Scheme 3).

**Figure 11:**
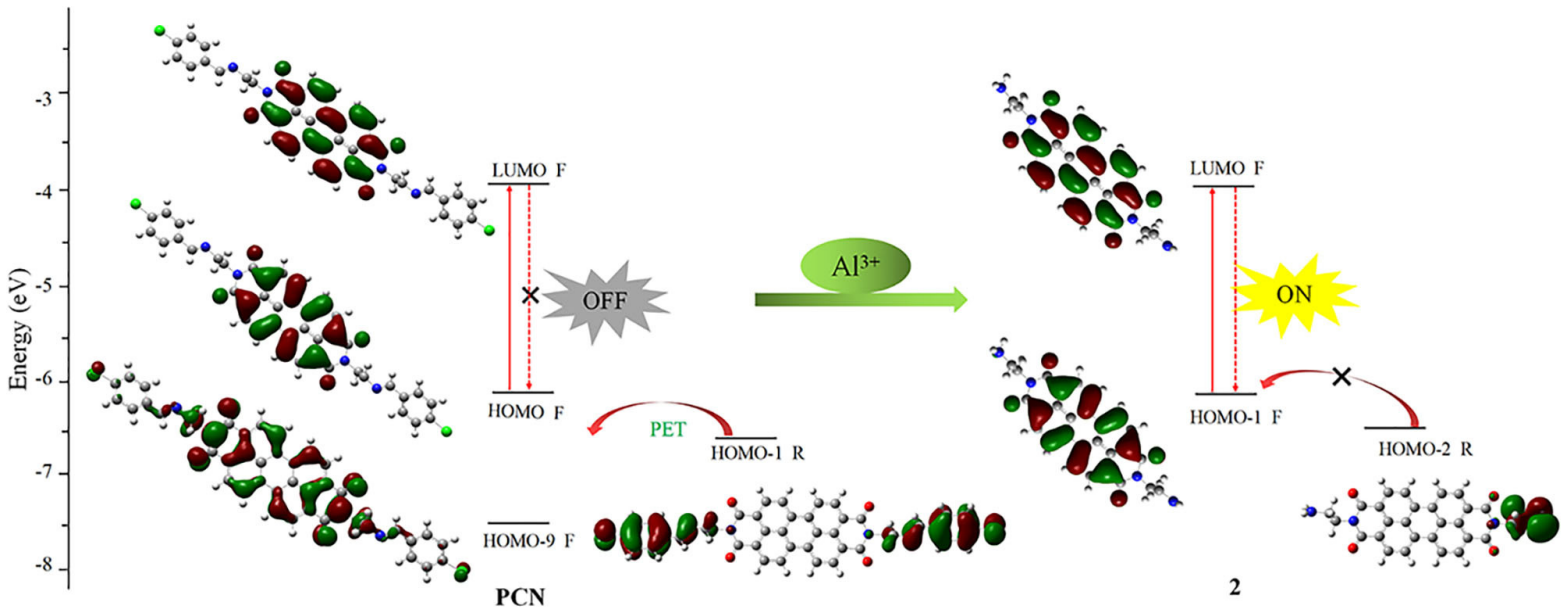
Frontier orbital energy diagram and electron transfer path in **PCN** and compound **2** (F, fluorophore; R, receptor segment).

In order to understand the sensing mechanism in the probe **PCN** in the absence or presence of Al^3+^ ion, density functional theory (DFT) quantum mechanical approach was performed. A Gaussian program (Frisch et al., 2009) was employed for DFT calculations at the B3LYP-D3BJ/def2-SV(P) level (Stephens et al., 1994; Weigend and Ahlrichs, 2005; Frisch et al., 2009; Grimme et al., 2010). The S1 state geometry was optimized, and the corresponding molecular orbitals were recorded by isosurfaces with the isovalue at 0.02. In the excite state of **PCN**, HOMO-1 (−6.62 eV) of the receptor unit is close to fluorophore HOMO (−6.16 eV) and located above the fluorophore HOMO-9 (−7.67eV). Hence, the electron of the HOMO-1 will be transferred to fluorophore regime through the reductive PET mechanism (Maity et al., 2019; Dos Santos Carlos et al., 2020). Significantly, the fluorescent “off” state of **PCN** is observed. After Al^3+^ ion hydrolyzed the probe **PCN**, the HOMO-2 (−6.44 eV) energy levels of the primary amine are decreased than that of the fluorophore HOMO-1 (−6.18 eV), as also observed. Hence, PET could not efficiently operate from the HOMO-2 of the primary amine to the fluorophore's HOMO-1 upon the removal of imine moiety, resulting in the fluorescent “on” state (Figure 11). Thus, the present calculation demonstrated that the electron donor imine leads to a highly efficient PET process.

## Conclusion

In summary, a novel PDI-based Schiff base derivative **PCN** was synthesized and utilized as a fluorescent probe. **PCN** exhibited a selective turn-on response to Al^3+^ over other coexisting competitive metal ions in DMF. DFT calculations showed that coordination of **PCN** to Al^3+^ inhibits the PET process. The C=N of **PCN** was hydrolyzed by Al^3+^, leading to the return to the intermediate compound, which resulted naked-eye visible color changes from colorless to yellow and nonfluorescent to yellow fluorescent. The detection limit was sufficiently low to determine the micromolar levels of Al^3+^. This sensor is valuable for Al^3+^ analysis in environmental samples.

## Data Availability Statement

The original contributions presented in the study are included in the article/Supplementary Material, further inquiries can be directed to the corresponding author/s.

## Author Contributions

YL and SG constructed the workflow, performed the data analysis, and wrote the manuscript. X-ML synthesized and purified the compound. LY and Y-LL contributed to data analysis. FY and YF contributed to the conception of the study, revised the manuscript, and approved the final version. All authors contributed to the article and approved the submitted version.

## Conflict of Interest

The authors declare that the research was conducted in the absence of any commercial or financial relationships that could be construed as a potential conflict of interest.
