# Supplementary material for: A Highly Selective Perylenediimide-Based Chemosensor: “Naked-Eye” Colorimetric and Fluorescent Turn-On Recognition for Al3+
Source: Front Chem. 2020 Sep 11;8:702. doi: 10.3389/fchem.2020.00702 (PMC7516037; doi:10.3389/fchem.2020.00702)
Supplement: Supplementary file 1 [file Data_Sheet_1.docx]

Supplementary Material

# Supplementary Figures


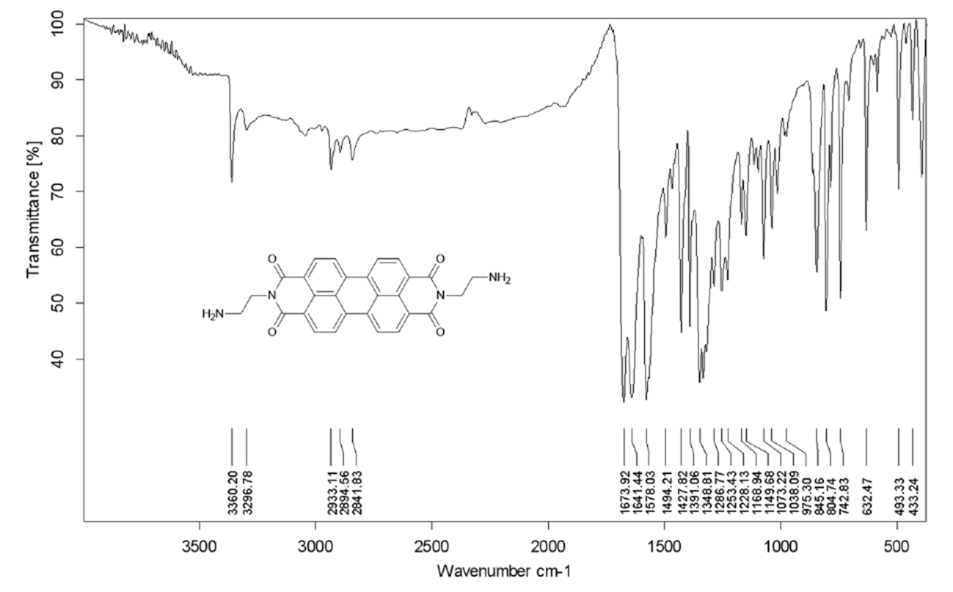


**Supplementary Figure 1.** IR spectrum of compound **2**.

**
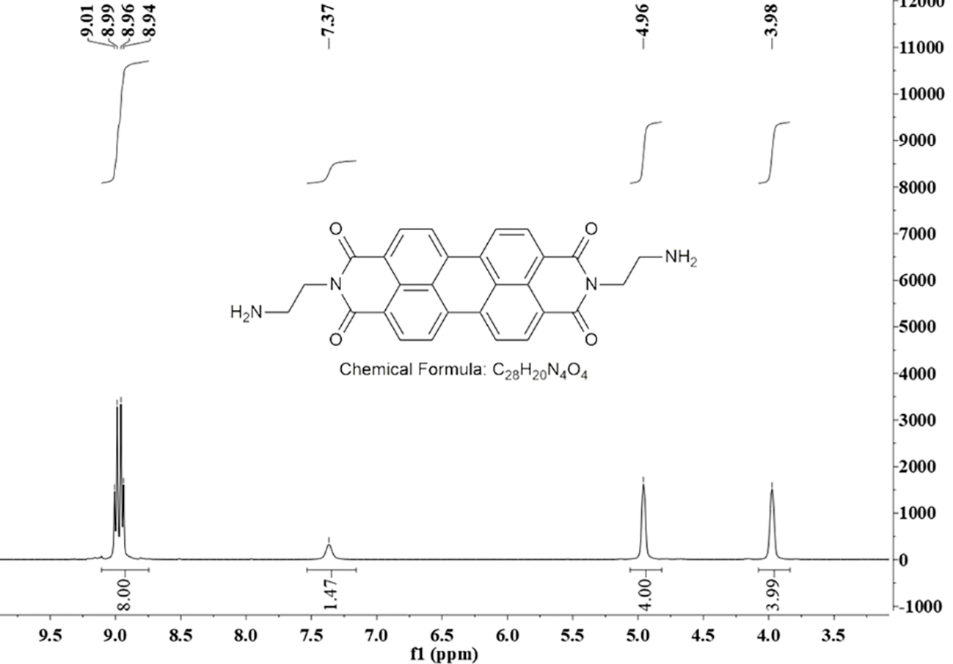
**

**Supplementary Figure 2.** ^1^H NMR spectra of compound **2**.

**
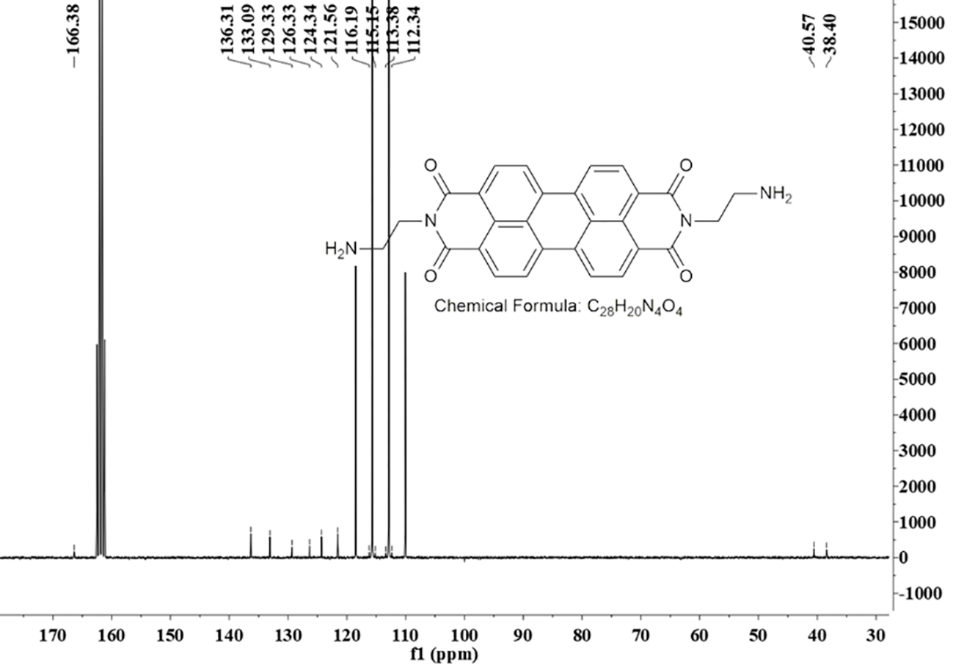
**

**Supplementary Figure 3.** ^1^C NMR spectra of compound **2**.


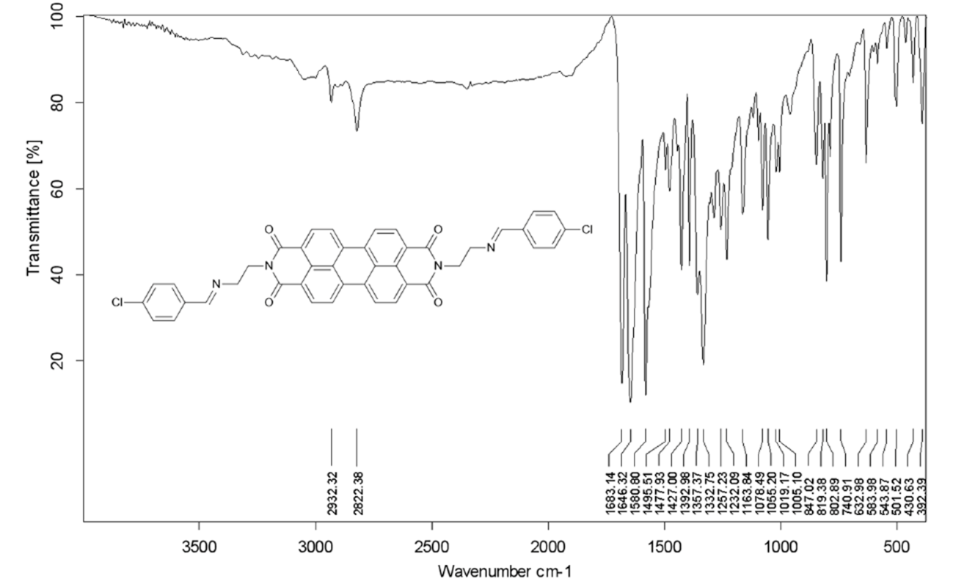


**Supplementary Figure 4.** IR spectrum of **PCN**.

**
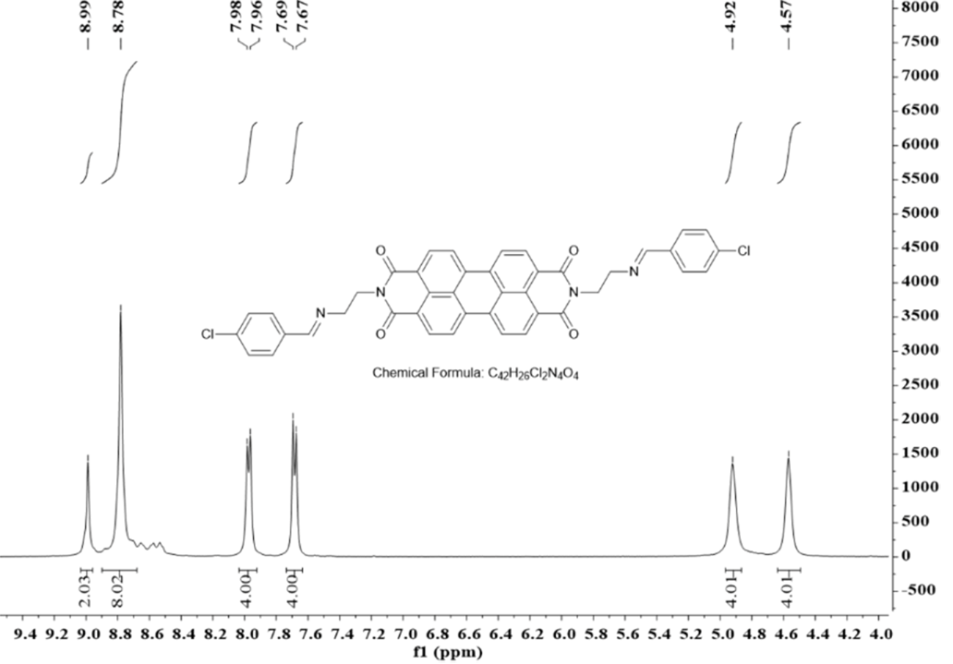
**

**Supplementary Figure 5.** ^1^H NMR spectra of **PCN**.

**
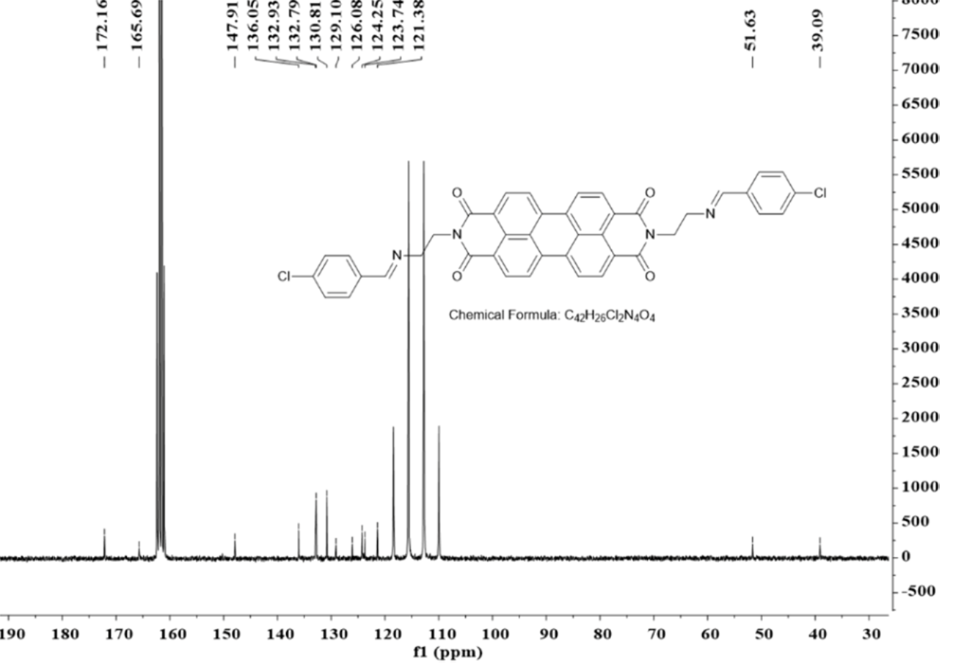
**

**Supplementary Figure 6.** ^1^C NMR spectra of **PCN**.


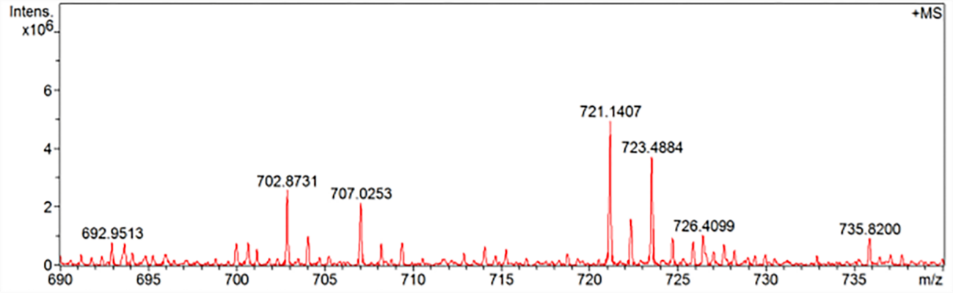


**Supplementary Figure 7.** HRMS of Compound **PCN**.

**
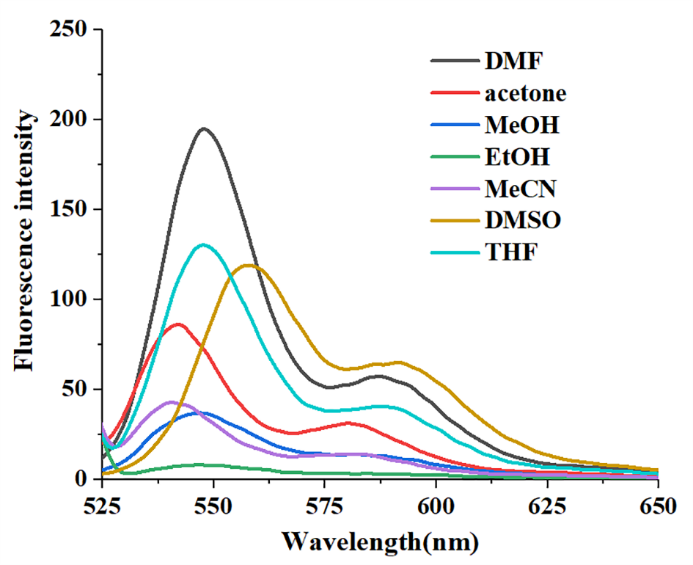
**

**Supplementary Figure 8**. Fluorescence spectra of **PCN** in different solvents.
